# Acceptability and factors associated with minimum postpartum stay in public health facilities among mothers in Mekelle: a mixed-methods study

**DOI:** 10.1186/s12884-025-08242-3

**Published:** 2025-10-08

**Authors:** Gebretsadik Kiros Lema, Haftamu Ebuy, Ytbarek Tadesse, Mache Tsadik

**Affiliations:** 1 School of Public Health, College of Health Sciences, Mekelle University, Mekelle, Ethiopia; 2 Department of Obstetrics and Gynecology, School of Medicine, College of Health Sciences, Mekelle University, Mekelle, Ethiopia

**Keywords:** Acceptability, Postpartum stay, Postnatal care, Mek’ele

## Abstract

**Background:**

Postnatal care in Ethiopia is often neglected. The WHO recommends mothers stay at health facilities for at least 24 hours after delivery, but information on the acceptability is lacking. This study aimed to assess the acceptability and factors associated with minimum postpartum stay at public health facilities among mothers in Mekelle in 2020.

**Method:**

A mixed, facility-based cross-sectional study design was employed among 604 mothers from May 14 to June 21, 2020. A multistage sampling technique was used to select study participants, while purposive sampling was used to recruit interviewees. Data were collected using Open Data Kit and exported to SPSS version 25.0 for analysis. In the multivariable regression, statistical significance was declared at a 95% confidence interval and a p-value < 0.05. Qualitative data were coded, categorized, and analyzed thematically.

**Results:**

A total of 620 postpartum mothers were enrolled, with 604 responding (97.4% response rate). Of the 604 participants, 527 (87.3%) mothers accepted the minimum recommended postpartum stay. Marital status (AOR = 2.70, 95% CI: 1.14–6.39), pulse rate assessment (AOR = 4.44, 95% CI: 1.34–14.68), confidentiality maintained by health care providers (AOR = 3.03, 95% CI: 1.61–5.69), satisfactory visiting hours for attendants (AOR = 2.48, 95% CI: 1.16–5.29), and mothers’ active participation in care (AOR = 4.61, 95% CI: 1.40–15.15) were identified as independent predictors of acceptability of the minimum postpartum stay. Most interviewees had a positive perception of the minimum postpartum stay.

**Conclusion:**

Acceptance of minimum postpartum stay was high, though actual stays fell short of WHO recommendations. Predictors of acceptability included marital status, pulse rate assessment, confidentiality, visiting hours, and active mother’s participation. Interventions to improve facility readiness and family awareness are essential to support optimal postpartum care duration. Further research is needed to explore the effects of early discharge on maternal and newborn health.

## Background

Postnatal care provides a supportive environment for a mother, her baby, and family to start their new life together. It includes routine clinical exams of the mother and infant, infant screening, feeding support, and ongoing information and assistance [1]. WHO recommends that after an uncomplicated vaginal birth in a health facility, mothers and newborns stay for at least 24 h. During this time, regular assessments of vaginal bleeding, uterine contraction, fundal height, temperature, and pulse should be conducted, with blood pressure measured shortly after birth and within six hours [2]. Significant changes occur in the postnatal period affecting mother and newborn well-being; thus, bathing should be delayed for at least 24 h, or six hours if cultural practices prevent this. Mothers and babies should remain together in the same room continuously during this period [2, 3].

The Sustainable Development Goals (SDGs) aim to reduce global maternal mortality to under 70 per 100,000 live births and end preventable deaths of newborns and children under five by 2030. Targets include neonatal mortality below 12 and under-five mortality below 25 per 1,000 live births. Achieving these goals requires quality care during labor, birth, and the immediate postnatal period [4].

Globally, an estimated 303,000 maternal deaths occur annually, with 99% in developing countries, over 60% in sub-Saharan Africa [5]. Ethiopia is among six sub-Saharan countries with a high maternal mortality ratio of 412 per 100,000 live births [6]. Most maternal deaths occur during labor and the immediate postpartum period, primarily due to hemorrhage (25%), pre-eclampsia/eclampsia (16%), and infection (10%) [7]. Hemorrhage, especially postpartum hemorrhage (PPH), is the leading cause, responsible for 27.1% of maternal deaths worldwide [8, 9], with over two-thirds linked to PPH defined as blood loss exceeding 500 ml within 24 h of birth [10]. PPH accounts for 32% of maternal deaths in northern Africa and 15.2% in sub-Saharan Africa [11].

Ethiopia has made notable strides in child health, yet mortality rates remain high. The 2016 EDHS reported neonatal and infant mortality rates of 29 and 48 per 1,000 live births, respectively, with leading causes like birth asphyxia (33%), preterm complications (26%), and sepsis (21%), largely preventable with better care around childbirth [6, 12].

Postnatal care has been described as a neglected area of maternity care in Ethiopia. The national coverage is at just 17%. In Tigray, while 90% of mothers attend at least one antenatal visit, only 42% receive postnatal care [6, 13]. Limited information exists on the acceptability of minimum postpartum stay and its associated factors at health facilities. This study aimed to assess the acceptability and determinants of minimum postpartum stay among mothers at public health facilities in Mekelle, while also exploring the perceptions of mothers and healthcare providers.

## Methods

### Study area and period

The study was conducted in Mekelle, the capital of Tigray, northern Ethiopia, approximately 783 km from Addis Ababa, between May 14 and June 21, 2020.

### Study design and population

A mixed, facility-based cross-sectional study design was used. The quantitative component included mothers who gave birth in selected health facilities and were transferred to postpartum care. The qualitative component involved purposively selected mothers, administrative staff, and healthcare providers. Exclusion criteria were cesarean delivery, stillbirth, severe illness or complications, and communication difficulties (e.g., mental disorders).

### Sample size determination and sampling procedures

The sample size was calculated using a single population proportion formula, assuming a 95% confidence level, 5% margin of error, 10% non-response rate, and a PNC coverage of 42% [6] in Tigray. With a design effect of 1.5, the final sample size was 620.

**Fig. 1:**
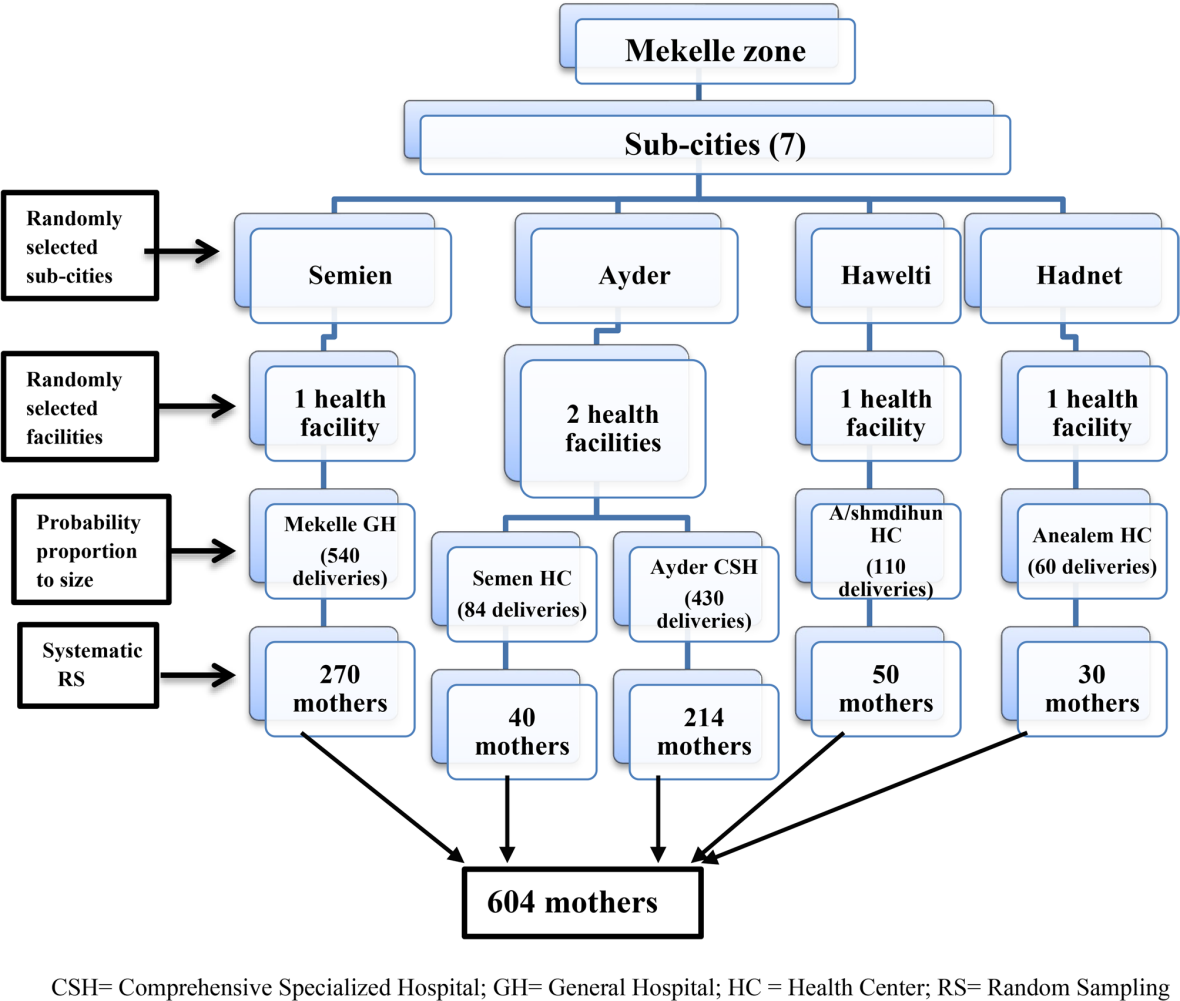
Schematic representation of sampling procedure applied to reach the study participants, May 14 –June 21, 2020

A multistage sampling technique was used: four sub-cities were randomly selected from seven, followed by random selection of public facilities within them. The sample size was proportionally allocated based on each facility’s average monthly deliveries. Participants were then chosen using systematic random sampling with a kth value of two (Fig. 1**)**.

For qualitative, purposive sampling was used for 15 key informant and in-depth interviews [5 mothers, 6 skilled birth attendants, and 4 administrative personnel] conducted until data saturation.

### Data collection tools and procedures

The questionnaire was developed through a review of relevant national and international literature [2, 14–16]. Structured and semi-structured questionnaires were developed in English, translated into Tigrigna, and back-translated to ensure consistency. They covered socio-demographic and economic data, obstetric history, and perceptions of mothers and health workers on minimum postpartum stay. After pre-testing, data were collected using interviewer-administered Open Data Kit (ODK) by a trained team of eight (one BSc midwife, three public health professionals, and four medical doctors), all fluent in Tigrigna. Three supervisors and the principal investigator monitored daily collection, discarding incomplete questionnaires.

For qualitative data, interview guides for key informant and in-depth interviews were prepared in English, translated into Tigrigna, and used by the investigator. Interviews were audio-recorded, transcribed, and translated into English.

### Data quality assurance

Two-day training prepared data collectors and supervisors on study objectives, tools, and procedures. The questionnaire was pre-tested on 5% of the target population at a similar facility outside the study area and revised accordingly. Regular supervision ensured data quality during collection.

### Data processing and analysis

Data were analyzed using SPSS version 25.0. Descriptive statistics (frequencies and percentages) summarized the study population. Variables with *p* < 0.25 in bivariate logistic regression were included in the multivariable model, where associations were considered significant at *p* < 0.05 with 95% confidence intervals. Multicollinearity was assessed using variance inflation factors (VIF), and model fit was evaluated with the Hosmer-Lemeshow test. Qualitative data were coded, categorized, and thematically analyzed using ATLAS.ti software.

### Operational or standard definitions

#### Acceptability

Mother’s verbal consent to stay at health facility within 6 to 24 h of delivery of placenta after they counseled about postpartum stay [17].

#### Minimum postpartum stay

A stay of at least 24 h of postpartum mother after giving birth at health facilities [2].

#### Postnatal services

Refer to the care provided to women after childbirth. It includes physical examinations, immunizations, family planning, health education on maternal and newborn care, treatment, and counseling [18].

## Result

### Socio-demographic characteristics of the participants

A total of 620 postpartum mothers were enrolled, with 604 responding (97.4% response rate). Participants’ ages ranged from 15 to 43 years, with most (55.6%, *n* = 336) aged 25–34 years. The mean age was 27.59 years (SD ± 5.35). Most mothers were Orthodox Christians (89.1%, *n* = 538) and urban residents (91.9%, *n* = 555) (Table 1).

**Table 1:** Socio-demographic characteristics of postpartum mother who stayed at public health facilities in Mekelle City Tigray, Ethiopia, 2020 (*n* = 604)

Variable	Category	Frequency	Percent (%)
Mother’s age in years	15–24	187	31.0
	25–34	336	55.6
	35–49	81	13.4
Religion	Orthodox Tewahido	538	89.1
	Muslim	57	9.4
	other (Catholic, protestant)	9	1.5
Residence	Urban	555	91.9
	Rural	49	8.1
Mother’s educational status	Unable to read and write	51	8.4
	Primary education	164	27.2
	Secondary education & above	389	64.4
Mother’s occupation^*^	Government employee	83	13.7
	House wife	304	50.3
	Other (Daily laborer, merchant, farmer.)	217	36
Marital status^*^	Married	557	92.2
	Other (Divorced, separated)	47	7.8
Husband’s education^*^ (*n* = 557)	Secondary education & above	363	65.2
	Other (can’t read & write, 1^o^ education)	194	34.8
Husband’s occupation^*^ (*n* = 557)	Government employee	136	24.4
	Private employee	134	24.1
	Merchant	121	21.7
	other (Daily laborer, farmer, jobless)	166	29.8
Monthly income in Ethiopian Birr	500–1500	53	8.8
	1501–2500	49	8.1
	> 2500	502	83.1
Home to health facility in minutes	2–30	495	82
	31–60	109	18
Did you get help to come to the health facility?	Yes	469	77.6
	No	135	22.4

^*^Due to the small number of participants in some standard education, marital status and occupation categories, we combined into ‘other’ to ensure adequate statistical power

### Knowledge related factors

The majority of mothers, 473 (78.3%), had heard about postpartum stay. A total of 534 (88.4%) knew at least one advantage of postpartum stay. Three-fifths (60.4%) were aware of the recommended duration, with 188 (51.5%) of them reporting 6 h after delivery of the placenta (Table 2).

**Table 2:** Knowledge related factors of postpartum mother who stayed at public health facilities in Mekelle City Tigray, Ethiopia, 2020 (*n* = 604)

Variable	Category	Frequency	Percent (%)
Ever heard about postpartum stay	Yes	473	78.3
	No	131	21.7
Knew at least one advantage of postpartum stay (*n* = 473)	Yes	418	88.4
	No	55	11.6
Knew the duration of Postpartum Stay	Yes	365	60.4
	No	239	39.6
Duration of postpartum stay mentioned (*n* = 365)	2–5 h.	14	3.8
	6 h.	188	51.5
	7–20 h.	61	16.8
	24 h.	102	27.9
Knew postpartum stay service in governmental health facilities is free of charge.	Yes	536	88.7
	No	68	11.3
Told you about postpartum stay by health care provider during ANC visit	Yes	387	64.1
	No	217	35.9
Told you about postpartum stay by health care provider during delivery	Yes	401	66.4
	No	203	33.6

### Obstetric & reproductive factors

Almost all mothers (98.8%) had ANC follow-up. Four hundred ninety-nine (83.6%) had four or more visits. Five hundred ninety-one (97.8%) mothers delivered in a health facility (Table 3).

**Table 3:** Obstetric & reproductive factors of postpartum mother who stayed at public health facilities in Mekelle City Tigray, Ethiopia, 2020 (*n* = 604)

Variable	Category	Frequency	Percent (%)
Gestational age in weeks	< 37 (Preterm)	19	3.1
	37–40 (Term)	456	75.5
	> 40 (Post-term)	129	21.4
Parity	Primi-para (1 birth)	227	37.6
	Multipara (2–4 birth)	296	49.0
	Grand multipara (> = 5 birth)	81	13.4
Pregnancy wanted	Yes	526	87.1
	No	78	12.9
ANC follow up	Yes	597	98.8
	No	7	1.2
Number of ANC visits (*n* = 597)	1–3	98	16.4
	>=4	499	83.6
Mode of delivery	Spontaneous vaginal delivery	599	99.2
	Instrumental delivery	5	0.8
Place of delivery	Health facility	591	97.8
	Home	13	9.3

### Health system factors

Most mothers (94%, *n* = 568) reported receiving a warm welcome from health care providers from reception to discharge. Additionally, 417 (69%) reported that their information was kept confidential, and 527 (87.3%) were satisfied with attendant visiting hours (Table 4).

**Table 4:** Health system factors of postpartum mother who stayed at public health facilities in Mekelle City Tigray, Ethiopia, 2020 (*n* = 604)

Variable	Category	Frequency	Percent (%)
HCP greet the mother	Yes	568	94.0
	No	36	6.0
HCP kept confidentiality of mother’s information	Yes	417	69.0
	No	187	31.0
HCP showed respect	Yes	552	91.4
	No	52	8.6
Satisfactory attendant visiting hour	Yes	527	87.3
	No	77	12.7
Mother’s active participation in care	Yes	572	94.7
	No	32	5.3

### Acceptability of minimum postpartum stay

Most mothers, 527 (87.3%), accepted the minimum postpartum stay. However, only three mothers (0.5%) stayed for 24 h after delivery of the placenta.

### Factors associated with acceptability of minimum postpartum stay

Variables with *p* < 0.25 in bivariate analysis were included in the multivariable logistic regression. Marital status, pulse rate assessment, confidentiality, visiting hours, and mother’s active participation were independent predictors of minimum postpartum stay acceptability. Married mothers had 2.7 times higher odds of acceptance (AOR = 2.70, 95% CI: 1.14–6.39); those with pulse rate assessed, 4.4 times (AOR = 4.44, 95% CI: 1.34–14.68); those reporting confidentiality, 3 times (AOR = 3.03, 95% CI: 1.61–5.69); mothers satisfied with visiting hours, 2.48 times (AOR = 2.48, 95% CI: 1.16–5.29); and those actively participating in care, 4.61 times (AOR = 4.61, 95% CI: 1.40–15.15) (Table 5).

**Table 5:** Factors associated with acceptability of minimum postpartum stay among postpartum mothers in Mekelle public health facilities, May – June 2020 (*n* = 604)

Variables	Category	Acceptability		COR(95%CI)	AOR(95%CI)
		**Yes**(*n* = 527) (87.3%)	**No**(*n* = 77) (12.7%)		
Marital status	Married	491 (88.2%)	66 (11.8%)	2.27 (1.10, 4.68)*	**2.70 (1.14**, 6.39)*
	other (Divorced, separated)	36 (76.6%)	11 (23.4%)	1	
During ANC visits, counseled about PPS	Yes	355 (91.7%)	32 (8.3%)	2.9 (1.78, 4.73)**	0.36 (0.12, 1.05)
	No	172 (79.3%)	45 (20.7%)	1	
During delivery, counseled about PPS	Yes	368 (91.8%)	33 (8.2%)	3.08 (1.89, 5.02)**	1.03 (0.38, 2.73)
	No	159 (78.3%)	44 (21.7%)	1	
Body temperature assessment	Yes	435 (89.5%)	51 (10.5%)	2.41 (1.43, 4.06)**	0.90 (0.45, 1.77)
	No	92 (78%)	26 (22%)	1	
Pulse rate assessment	Yes	501 (88%)	68 (12%)	2.55 (1.15, 5.67)*	**4.44 (1.34**, 14.68)*
	No	26 (74.3%)	9(25.7%)	1	
Uterine contraction assessment	Yes	283 (83.7%)	55 (16.3%)	0.46 (0.28, 0.78)*	0.85 (0.39, 1.88)
	No	244 (91.7%)	22 (8.3%)	1	
Urine void assessment	Yes	223 (93.3%)	16 (6.7%)	2.79 (1.57, 4.98)**	0.66 (0.25, 1.71)
	No	304 (83.3%)	61 (16.7%)	1	
Counseled about EBF	Yes	376 (93.5%)	26 (6.5%)	4.88 (2.94, 8.12)**	2.16 (0.65, 7.17)
	No	151 (74.8%)	51 (25.2%)	1	
Counseled about FP	Yes	355 (93.7%)	24 (6.3%)	4.55 (2.72, 7.63)**	1.39 (0.34, 5.73)
	No	172 (76.4%)	53 (23.6%)	1	
Counseled on HIV transmission	Yes	355 (93.2%)	26 (6.8%)	4.04 (2.44, 6.71)**	1.54 (0.62, 3.80)
	No	172 (77.1%)	51 (22.8%)	1	
Counseled on child care	Yes	368 (93.2%)	27 (6.8%)	4.28 (2.59, 7.09)**	1.01 (0.28, 3.65)
	No	159 (76.1%)	50 (23.9%)	1	
Counseled on child danger signs	Yes	362 (93.3%)	26 (6.7%)	4.30 (2.59, 7.14)**	1.47 (0.52, 4.14)
	No	165 (76.4%)	51 (23.6%)	1	
Counseled on personal hygiene	Yes	356 (92.7%)	28 (7.3%)	3.64 (2.21, 6.00)**	1.56 (0.59, 4.11)
	No	171 (77.7%)	49 (22.3%)	1	
HCP greet you	Yes	502 (88.4%)	66 (11.6%)	3.34 (1.57, 7.11)*	0.83 (0.16, 4.15)
	No	25 (69.4%)	11 (30.6%)	1	
HCP kept confidentiality your information	Yes	388 (93%)	29 (7%)	4.62 (2.80, 7.61)**	**3.03 (1.61**, 5.69)**
	No	139 (74.3%)	48 (25.7%)	1	
HCP respect	Yes	487 (88.2%)	65 (11.8%)	2.24 (1.12, 4.50)*	0.43 (0.09, 1.89)
	No	40 (76.9%)	12 (23.1%)	1	
Satisfactory attendant visiting hour	Yes	474 (89.9%)	53 (10.1%)	4.05 (2.31, 7.08)**	**2.48 (1.16**, 5.29)*
	No	53 (68.8%)	24 (31.2%)	1	
Mother’s active participation in care	Yes	508 (88.8%)	64 (11.2%)	5.43 (2.56, 11.51)**	**4.61 (1.40**, 15.15)*
	No	19 (59.4%)	13 (40.6%)	1	

*1* Reference, *AOR* Adjusted Odds Ratio, *COR* Crude Odds Ratio, *C.I* Confidence Interval

Significant at * (p < 0.05), ** (p < = 0.001)

### Responses of participants

Key informant and in-depth interviews were conducted at Quiha Hospital, Ayine-Alem, Mekelle, and Kasech Health Centers to complement the quantitative data. Qualitative responses were organized into four themes:

### Theme 1: Adherence of PNC guidelines

All participants in the key informant interviews demonstrated positive adherence to the PNC guidelines from the World Health Organization, as well as national and regional guidelines. One of the interviewees stated:

“*…We are using BEmONC guideline. The guideline says that mothers should stay 24 hours but due to shortage of beds and rooms sometimes mothers are discharged from 6–12 hours after delivery…*” (26 years old, HCW).

In harmony with the above evidence another health care workers stated that:

*“…We are using the WHO guideline for postnatal service. Also we have Ethiopian guidelines and we are using both of them for prenatal and postnatal service. We are working to stay mothers for 24 hours but most of the time they go home before 24 hours because we have shortage of beds…”* (30 years old, HCW, Medical director).

“…*When mothers stay here (health facility) they are followed every 15 minutes for the first 2 hours to check bleeding*,* BP*,* Pulse Rate and RR. Then the follow up continues every 30 minutes for the next 2 hours to check the above parameters…*” (26 years old, HCW).

In agreement with the health care workers, 29 years old postpartum mother was also explained the situation:

*“…The services I get here (health facility) until now are they measured my heart beat*,* blood pressure and they asked me about bleeding also they observed me for any problem. But they didn’t measure me temperature and urine output…”* (29 years old, postpartum mother).

### Theme 2: Perception towards acceptability of minimum postpartum stay

Most of participants had a positive perception towards acceptability of minimum postpartum stay at public health facilities. A health care worker stated:

*“…Mothers of course they will accept the minimum postpartum stay I mean 24 hours. Just it is matter of counseling. If the health personnel counseled them especially about the complication with preparedness and commitment*,* mothers will accept the stay…”* (29 years old, HCW).

In agreement with the Health care worker, postpartum mothers were stated their view for the minimum postpartum stay at health facilities like this:

*“…It’s better to stay at least 24 hours. I mean it’s better to go after checking everything. We don’t know what will happen it could be good or bad. So it’s better to stay here…”* (19 years old, postpartum mother).

*“…Staying 24 hours is good because it’s important for better and insured care mother and baby. It is better to stay here than going home. I wish I could go home even to deliver but I came here because it has benefit and I am happy…”* (24 years old, postpartum mother).

### Theme 3: Benefits for acceptability of minimum postpartum stay

Key informants and in-depth interview participants highlighted that informing and empowering mothers, families, and attendants about postpartum services are vital to promoting acceptance of the recommended stay. However, health professionals reported difficulties convincing mothers to remain the full 24 h after delivery. Consequently, some actions have been taken, as described by the key informants:

*“…At the time*,* we informed them (mothers) to stay 24 hours. There were challenges like the opinion 6 hours is enough after delivery. And then we were working in collaboration with women development army and other associations. After that*,* mothers and the community accepted to stay 24 hours…”* (36 years old, HCW).

In agreement with above findings 19 years old postpartum mother stated that:

*“…I stayed here for 12 hours. They informed me to stay 24 hours. In that case I refused the information. After that they called to my family and we discussed about it. Finally*,* we agreed. Until now my stay is good…”* (19 years old, postpartum mother).

Providing friendly services and allowing for cultural ceremonies were also important opportunities to increase acceptance of the minimum postpartum stay. In agreement with the health care workers, a 24-year-old postpartum mother in the in-depth interview explained:

“…*Until now (Stayed 8 hours) my stay is good. Staying at home and staying here (health facility) has difference. If something happen when I am at home I will be anxious about my health. But I am here they will follow and help me… For example if I have bleeding or my baby has cord bleeding*,* they will help me …even if they are working I can call them for help*…” (24 years old, postpartum mother).

*“…When I told them… I don’t have family… they (health care providers) said me that here (health facility) everybody is your brother*,* sister and parents. They told me we are here for you. It’s very good staying here…”* (24 years old, postpartum mother).

Another postpartum mother indicated:

*“…All my children (4 babies) were born at home. But this baby delivered here (Health facilities). It’s very good for health… giving birth here is good for me and my baby. Here its good.my family and neighbors are visiting me. Like home*,* here are also cultural ceremonies such as coffee and porridge…”* (33 years old, postpartum mother).

In bond with postpartum mothers, a health care provider stated:

*“…mothers are not complaining. We have coffee and porridge. Their families can prepare tea and coffee. And also we allowed 1–2 attendants to stay with them to decrease overcrowding…”* (31 years old, HCW).

### Theme 4: Barriers for acceptability of minimum postpartum stay

Resource limitations (such as shortages of beds, bathrooms, and toilets), family pressure, and cultural issues were major problems in all health facilities, especially in hospitals. One of the key informants said:

“… *Except limitation/shortage of beds we don’t have any reason that makes us to discharge early… Staying 24 hours is good for mothers. Because we don’t know what could happen within 24 hours. Sometimes after they go home there is bleeding*,* baby suffocation*,* baby may get breastfeeding difficulty and vomiting repeatedly. Even when they stay here there are such cases…*” (35 years old, HCW).

Similar evidence was also gathered from postpartum mother:

“…*staying here (postpartum room) is a benefit for me. Especially to get close follow up if there is some problems like bleeding. But it is difficult to stay 24 hours here without bath room and special toilet for a mother… if they (HCW) suspect anything they should tell me to come back again but it doesn’t make sense to seat here 24 hours without problem…*” (29 years old, postpartum mother).

During the in depth interview, another postpartum mother also stated:

*“…Staying 24 hours is too long. It’s not comfortable for family. Staying 6 hours is enough and good. Because my husband is living in another region and my children are alone*…” (37 years old, postpartum mother).

## Discussion

This study aimed to assess the acceptability and factors associated with minimum postpartum stay in public health facilities. In this study, the overall acceptability of minimum postpartum stay at health facilities among the study population was 87.3%. This finding is similar to studies conducted in Madagascar (87.5%) and Gambia (89.3%) [19]. The qualitative portion of this study also supported this finding, as most participants expressed positive views. However, the current study’s acceptability rate was higher than those reported in Burundi (43.8%), India (46.1%), Swaziland (26.6%), and Chad (41.2%) [19, 20]. This variation might be due to differences in sample size and study settings. On the other hand, the actual length of stay was much shorter (< 24 h) than the WHO recommendation of 24 h for uncomplicated vaginal deliveries [2].

Married women in this study had 2.7 times higher odds of accepting minimum postpartum stay compared to divorced or separated women. This significant association contrasts with a study conducted in Tigray, which found no association between marital status and postnatal service use [15]. This finding was supported by a postpartum mother interviewed during the qualitative portion, who mentioned that leaving children at home alone was a reason for not accepting postpartum stay.

This study showed that mothers who had their pulse rate assessed were 4.44 times more likely to accept the minimum postpartum stay than those who were not assessed. The qualitative findings highlighted that close follow-up and friendly service were important factors influencing mothers’ acceptance of the stay.

Mothers who reported that their confidentiality was maintained were 3.03 times more likely to accept the minimum postpartum stay than their counterparts. This finding is consistent with a study conducted by Asma Yunus and colleagues in Tribal areas, which found that the type of health care provider was significantly associated with timely postnatal checkups within the first 24 h [21]. This might be due to the trustworthiness of health care providers by the mothers.

Satisfactory attendant visiting hours were significantly associated with acceptability of minimum postpartum stay. Mothers who reported satisfactory visiting hours were 2.48 times more likely to accept the stay than those who did not. This finding aligns with the qualitative data, where participants mentioned that allowing cultural ceremonies for postpartum mothers and their families or attendants contributed to acceptance.

This study presented with the finding of mothers who had active participation in all services during their stay was 4.61 more likely to accept the minimum postpartum stay than their counter parts. This finding is supported by a study conducted in China, which indicated that service quality was strongly associated with postpartum care [22]. The qualitative data also reflected this, with interviewees explaining that family involvement and considering the mother’s opinion during decision-making encouraged acceptance of the stay.

### Limitation of the study

The main limitation of this facility-based cross-sectional study was the inability to establish a temporal relationship between exposure and outcome. Additionally, the findings may not fully represent the broader community.

## Conclusion

Acceptance of minimum postpartum stay was high, though actual stays fell short of WHO recommendations. Predictors of acceptability included marital status, pulse rate assessment, confidentiality, visiting hours, and active mother’s participation. Interventions to improve facility readiness and family awareness are essential to support optimal postpartum care duration. Further research is needed to explore the effects of early discharge on maternal and newborn health.

## Data Availability

The data sets used and/or analyzed in this study are available from the corresponding author upon reasonable request via email.

## Abbreviations

ANC: Antenatal Care
PNC: Post Natal Care
WHO: World Health Organization
